# RNA Unwinding by NS3 Helicase: A Statistical Approach

**DOI:** 10.1371/journal.pone.0006937

**Published:** 2009-09-22

**Authors:** Srikesh G. Arunajadai

**Affiliations:** Department of Biostatistics and Anesthesiology, Columbia University, New York, New York, United States of America; University of Edinburgh, United Kingdom

## Abstract

The study of double-stranded RNA unwinding by helicases is a problem of basic scientific interest. One such example is provided by studies on the hepatitis C virus (HCV) NS3 helicase using single molecule mechanical experiments. HCV currently infects nearly 3% of the world population and NS3 is a protein essential for viral genome replication. The objective of this study is to model the RNA unwinding mechanism based on previously published data and study its characteristics and their dependence on force, ATP and NS3 protein concentration. In this work, RNA unwinding by NS3 helicase is hypothesized to occur in a series of discrete steps and the steps themselves occurring in accordance with an underlying point process. A point process driven change point model is employed to model the RNA unwinding mechanism. The results are in large agreement with findings in previous studies. A gamma distribution based renewal process was found to model well the point process that drives the unwinding mechanism. The analysis suggests that the periods of constant extension observed during NS3 activity can indeed be classified into pauses and subpauses and that each depend on the ATP concentration. The step size is independent of external factors and seems to have a median value of 11.37 base pairs. The steps themselves are composed of a number of substeps with an average of about 4 substeps per step and an average substep size of about 3.7 base pairs. An interesting finding pertains to the stepping velocity. Our analysis indicates that stepping velocity may be of two kinds- a low and a high velocity.

## Introduction

The study of RNA unwinding by helicases is a problem of basic scientific interest. Recent advances in single molecule techniques have allowed the study of these proteins and their action at an unprecedented detail [1], [2]. The HCV RNA helicase NS3 is one such example. The Hepatitis C virus infects nearly 3% of the world population. NS3, as one of the viral-encoded proteins that are essential for viral genome replication, has been a potential target for therapeutic intervention. Several studies [3]–[5] have focused on the RNA unwinding mechanism using bulk measurement techniques. More recently, in [1] the RNA unwinding mechanism was studied by observing individual mechanistic cycles using optical tweezers. They employed Fourier analysis on the distribution of pairwise distances of the unwinding to study the unwinding characteristics. This work is motivated by these single molecule experiments [1]. We aim to perform a statistical analysis on the RNA unwinding data and compare with the conclusions reached before. The results presented here were obtained using the data analysis methods proposed in Arunajadai [6]. In this method, RNA unwinding by NS3 is hypothesized to occur in a series of discrete steps. In [1], Dumont et al. proposed the concept of pauses and subpauses, which are periods of constant extension between the discrete steps observed directly from single molecule experiments. Such behavior has also been observed in single molecular studies involving the separation of double-stranded DNA into two separated single strands at constant force [7], [8] which may provide information pertinent to the mechanism of DNA replication. Here the pauses are believed to caused by a series of energy minima where the strand separation halts and will not commence until the energy barrier is overcome. Even in identical molecules the number of base pairs that separate as function of time varies as separation requires random thermal activations that differ in different identical molecules. In this work, a classification algorithm is employed on the periods of constant extensions estimated by the statistical model. This analysis helps one to understand if such distinctions exist and if so, whether the differences are statistically significant. We see that there is reasonable agreement among most of the characteristics of the unwinding mechanism but there are also some new inferences which might be worth further investigation.

## Methods

### Experiment

**Figure 1:**
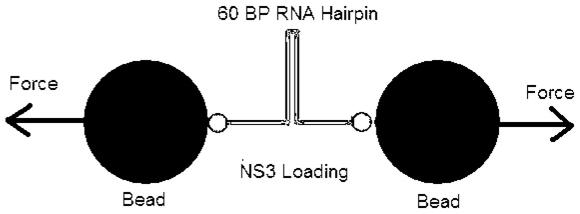
Schematic of the Experiment. A 60 BP RNA hairpin held between the beads of an optical tweezer.

Here we describe the experimental setup followed in [1] that was used to obtain unwinding trajectories of NS3 helicase in real time. The enzyme used is the full-length protein of the hepatitis C virus helicase (NS3) from HCV genotype 1a. The single molecule assay can directly follow the movement of NS3 on its RNA substrate. The RNA secondary structure used in this experiment is called the hairpin. Optical tweezers are used to apply a constant tension between the two beads attached to the ends of a 60 base-pair (bp) RNA hairpin. The end to end distance change of the RNA is recorded as it is unwound by NS3. In [1], it is shown that any unwinding of the RNA at external forces below 19 pN (pico Newton, 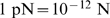) must be helicase catalyzed. Thus the double stranded RNA is held by the optical tweezers at a constant force ranging between 5 and 17 pN. NS3 and ATP were flown together in a buffer. As NS3 unwinds the hairpin, the distance between the beads increase so as to maintain a constant force on the molecule. For each pair of base-pair unwound a pair of nucleotides (nt) is released. A schematic of the experiment is shown in figure 1. For the technical details of the experiment refer to [1]. The extension of the unwound RNA is recorded in nanometers (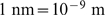). The data for this study was provided by Dr. Wei Cheng, of the Bustamante Lab, University of California Berkeley. The details of the experiment, data collection and the original analysis can be found in [1].

### Statistical Analysis

It is hypothesized that the NS3 helicase enzyme unwinds RNA in a series of discrete steps. The key questions of interest from a biological perspective are

1. What is the size of these steps in base-pairs?
2. Are there substeps within these steps? If so how many substeps are there in a given step and what is the size of these substeps?
3. Is there a pause duration i.e. a period of constant extension between these steps?
4. Are there subpauses between the substeps?
5. What is the stepping velocity of these steps?

Dumont et.al. in [1], perform a Fourier analysis on the distribution of pairwise distances from a single trace and compute the step size as frequency with the highest power. The steps were then detected by scanning for the maximum slope within a running-window. Pause durations were defined as the intervals between two steps and the velocity as the slope from a linear fit. Substeps were then detected using a running-window within a step. Subpauses i.e. periods between substeps were required to be longer than 80 ms. Thus we see that the analysis depends on setting certain conditions like requiring the user to choose an appropriate running window size. In this work we avoid the above assumptions but make assumptions on a random process that will be used to model the unwinding mechanism. The assumptions made in this work regarding the random process are

1. The unwinding occurs in a series of discrete steps.
2. The step locations themselves occur in accordance to an underlying point process.
3. The process is accompanied by stationary noise (by stationary we mean that the mean and covariance structure of the noise data is time invariant).

The above framework for the unwinding mechanism is analyzed using the methodology described in Arunajadai [6] which employs methods pertaining to the following two areas of statistics:

1. 79Change Point Problems: Consider a random process where certain distributional characteristic of the process (for example, the mean) changes at certain points in time. Such class of problems are referred to as change point problems. References [9], [10] provide a comprehensive review of change point problems. In this work it is assumed that the mean level of the process (i.e. mean level of the step) 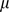 changes at certain time points in time while the variance 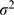 remains constant throughout the process. It is also assumed that each individual homogenous segment follows a normal distribution 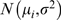 where 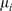 is the mean level of the 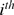 step.
2. Point Processes: Point processes are a type of random processes employed to study the collections of point occurrences. An important class of point processes is the renewal processes. Here it is assumed that the intervals between the point occurrences are independently and identically distributed. Here we consider the gamma distribution 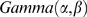 with shape parameter 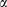 and scale parameter 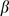, for the intervals between point occurrences. For 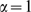, the Gamma distribution coincides with the exponential distribution yielding a Poisson process as a special case. For other values of 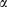 one gets sequences of points with more (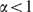) and less (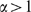) clustering. With the so called substeps (to be discussed below) occurring close together one might expect an (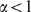) as opposed to 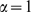 as assumed in [1] in their Poisson analysis. For a review of point processes refer [11]–[13].

**Figure 2:**
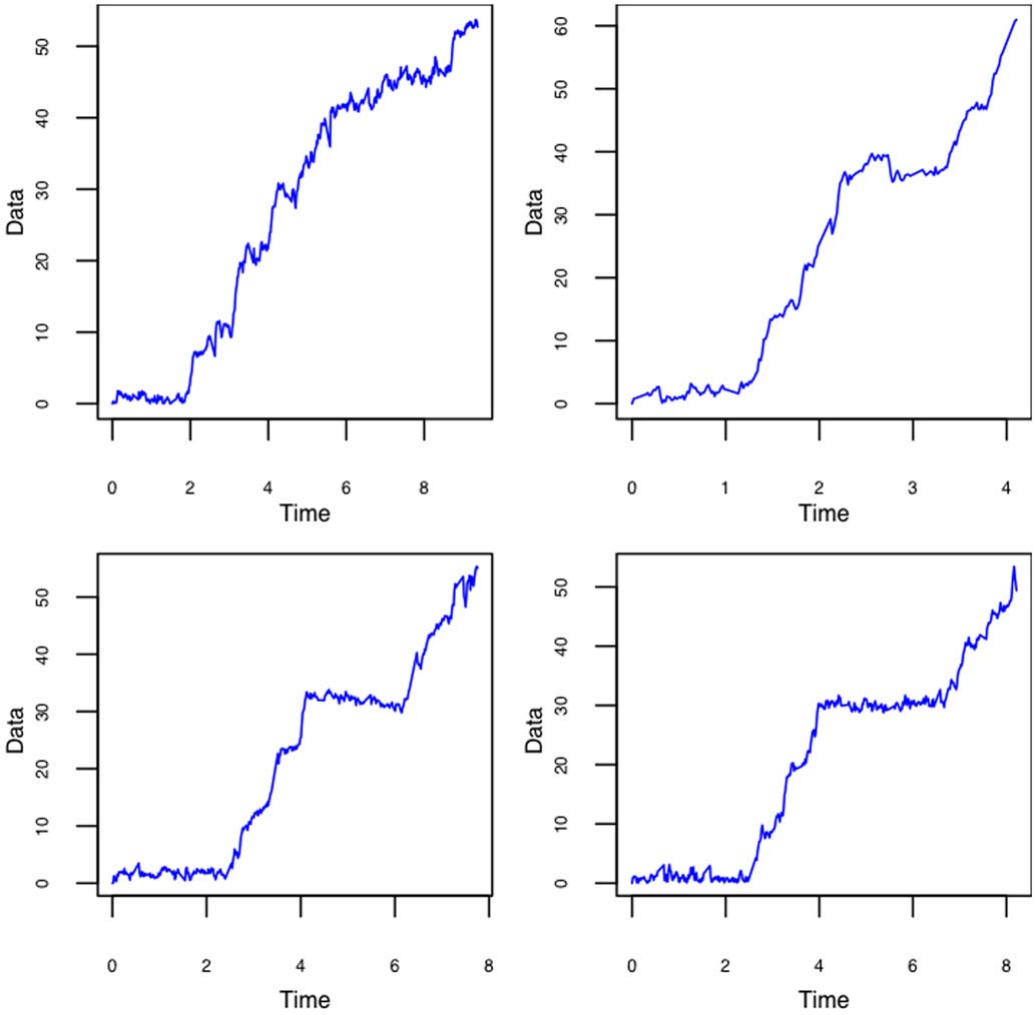
RNA unwinding trace for four sample data. Time is in ms and Extension in nm.

In the RNA unwinding by NS3 Helicase the steps are separated by pauses. In the DNA unzipping literature [7], [8] these pauses are believed to be caused by a series of energy minima where the strand separation stops until a certain energy threshold is overcome. Even in identical molecules the number of base pairs that separate as function of time varies as separation requires random thermal activations that differ in different identical molecules. These *random thermal activations* or its counterparts in the RNA unwinding are assumed to be a from a point process i.e. events occurring in a random way in time. To determine these point occurrences we employ change point models. That is at each of these point occurrences, the RNA unwinds and an extension is recorded as shown in the unwinding trace in figure 2. Thus when the RNA unwinds at the point occurrence, the mean level of the extension changes and the change point methodology is used to detect these mean level changes which in turn estimate the times of the point occurrences.

**Figure 3:**
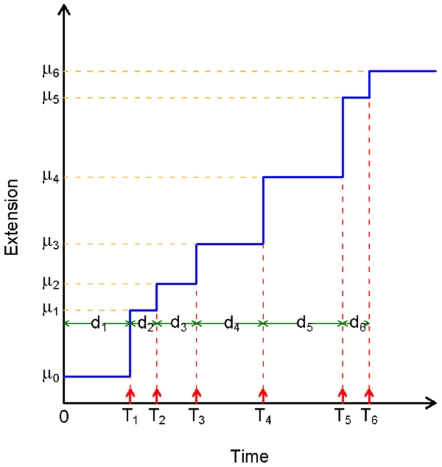
Schematic of the RNA unwinding process. 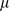
 represents the mean level of the steps and 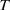 represents the jump locations. 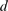 is the inter-jump interval.

Figure 3 shows the schematic of the point process driven change point model on which the RNA unwinding is modeled. Here 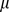 denotes the mean level of the step, 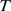 the time of the jump and 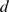 the interval between the steps. Note that the time of the jump 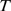 can be expressed as the sum of the inter-jump intervals 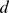 as
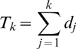
(1)


The mean level 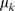 of step 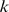 may be given as
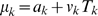
(2)where 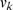 is the velocity of the step and 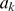 is the intercept term. Thus the observed value 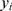 at time 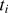 is given by
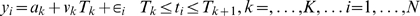
(3)where 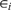 is the accompanying noise and is assumed to follow a normal distribution 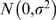, 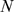 is the number of data points and 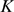 the number of steps. The Gamma distribution has the property that if 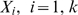 are independent and distributed as 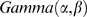 then 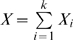 is distributed as 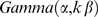. Assuming the intervals 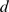 between the steps follows a gamma distribution 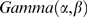 where 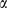 is the shape parameter and 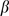 is the scale parameter, the distribution of the time points at which the steps occur follows from the above property of the gamma distribution and equation (1) and is given by
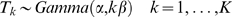
(4)Arunajadai [6] provide a robust-resistant approach and the associated algorithms to detect change points. The change point detection is based on the premise that the points in one step behave as outliers with respect to the distribution of the points in another step. A weighting procedure is described where points that behave as outliers are assigned zero weights. The paper also discusses the maximum likelihood approach to estimate the parameters 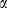, 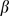 and 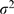.

## Results

### Model Fit and Diagnostics

**Figure 4:**
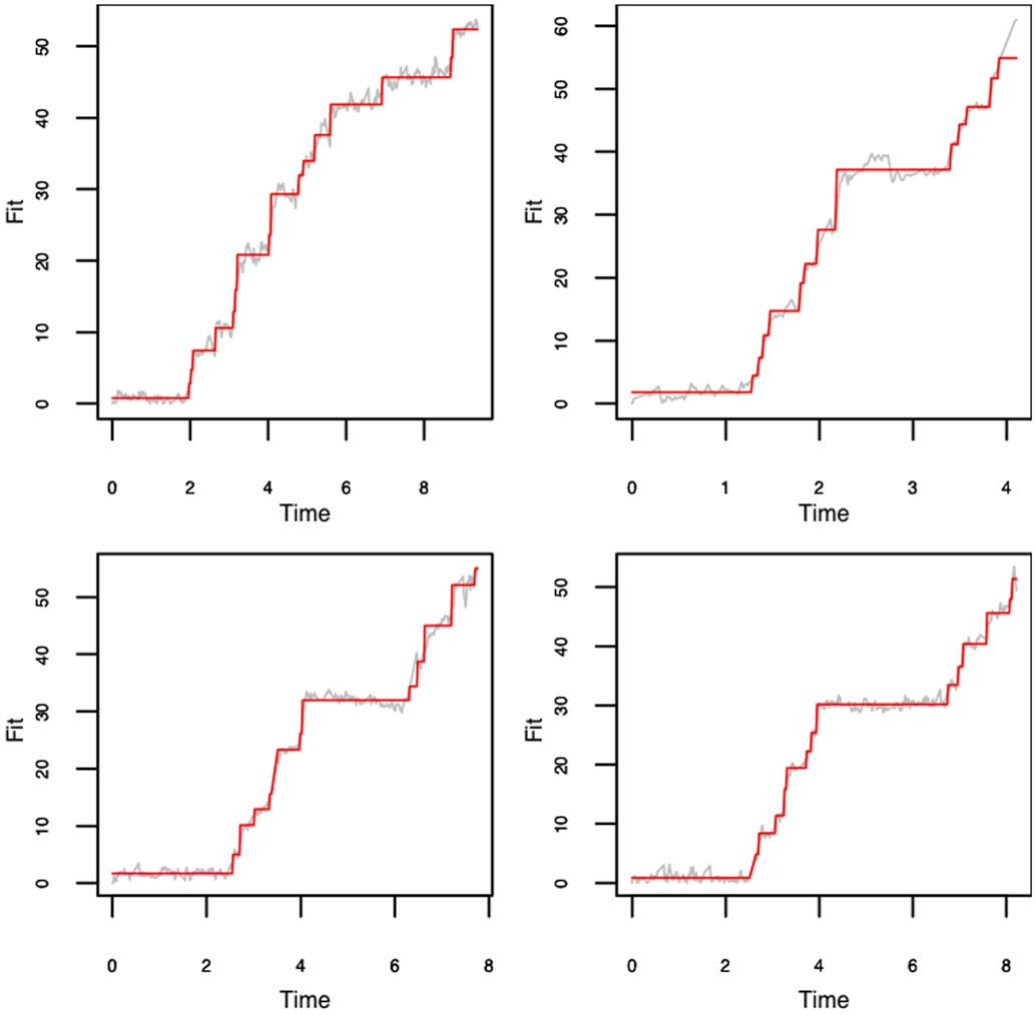
RNA unwinding model fit. the red lines are the fitted value superimposed on the gray lines indicating the actual data.

**Figure 5:**
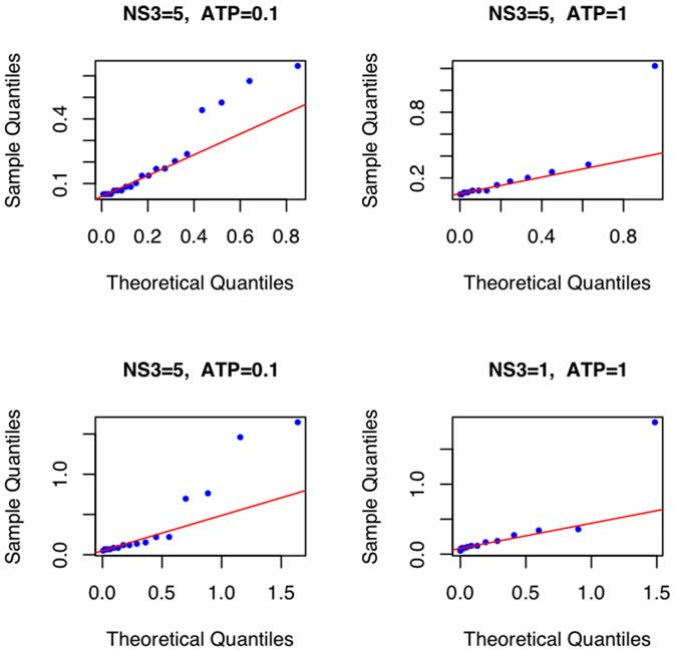
Jump Intervals - Gamma QQ Plot. The dots indicate the observed data and the red line indicates the reference line. NS3 and ATP concentrations are labeled above the plot.

Figure 2 shows the plot of the raw data and figure 4 shows the fitted values obtained from model described in equation (3) using the algorithms described in [6]. Recall that there are two assumptions imposed on equation (3). First, the intervals between the jumps have a gamma distribution with a common shape parameter 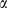 and scale parameter 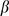, i.e. 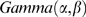. Second, the noise accompanying the unwinding process are independent with zero mean normal distribution 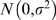. Figure 5 shows the Gamma quantile-quantile plot for the time intervals between jumps from the four fits in figure 4. Quantile-Quantile plots (QQ-plot) aid in validating the distributional assumption in that when points fall along the reference line (Line through the 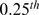 and 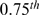 quantiles of the empirical and the theoretical distributions. The data falling along this line might indicate that the data has the theoretical distribution but possibly with different shape and scale parameter and hence the line need not have an intercept of zero and a slope of 1) the assumption can be taken to be valid. There are fewer outliers for the ATP concentration of 1 mM than for 0.1 mM. What these outliers mean and their dependence on ATP concentration will be discussed below. The plots otherwise seem to suggest that it is reasonable to assume that the time intervals between the jumps is gamma distributed.

**Figure 6:**
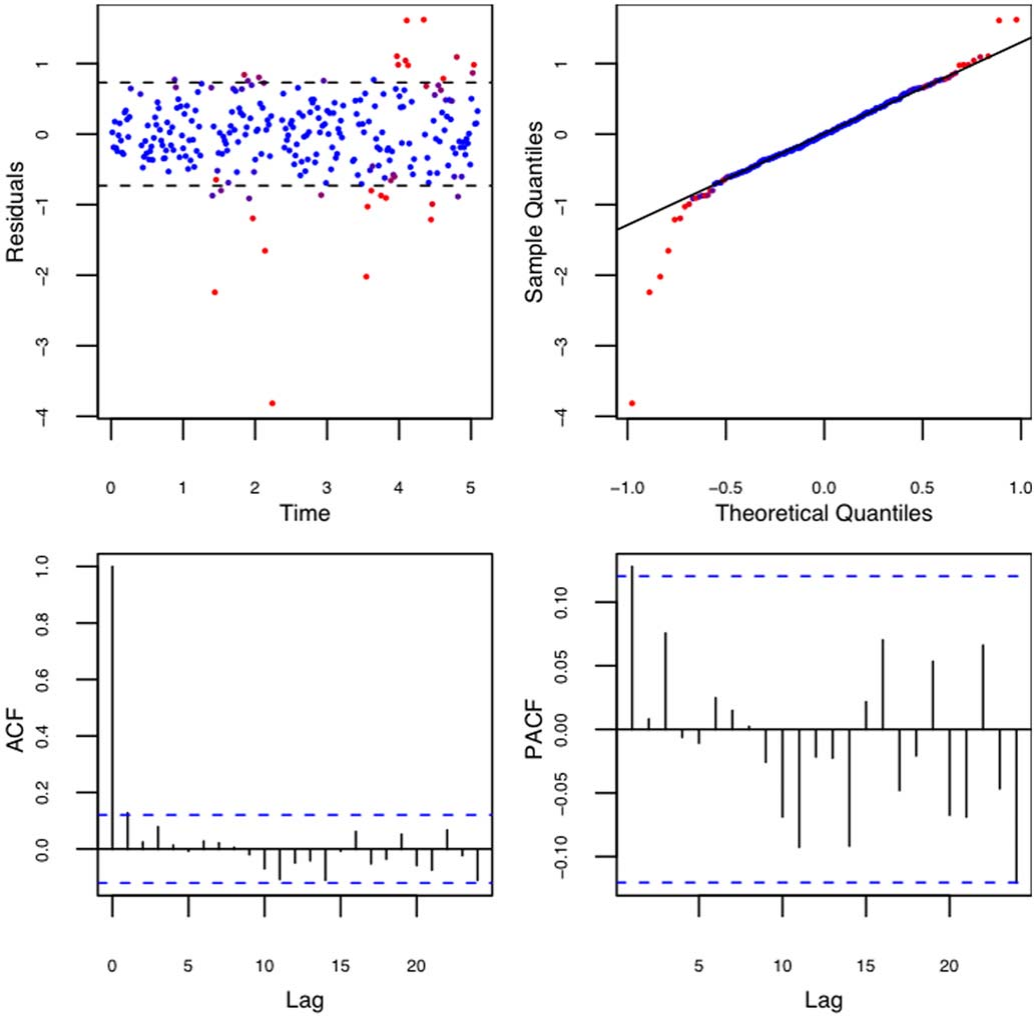
Residuals from fit. Residual plot, QQ-plot of residuals, the autocorrelation function (ACF) and the partial autocorrelation (PACF) plots of the residuals. The red dots indicate the points with zero weights and the blue dots non-zero weights.

The residuals from this fit are shown in figure 6. There seems to be no obvious pattern in the residual plot with time indicating that the residuals are randomly distributed across time. The QQ-plot shows that the only outlier data points i.e. that ones straying away from the reference line are the ones with zero weights (red dots). The plots (ACF) and (PACF) show the autocorrelation and the partial autocorrelation plots. The autocorrelation is the correlation between points at different lags and the partial autocorrelation is the correlation between points at different lags that is not accounted for by the intermediate lags. For more details see [14]. The plots indicate that the correlations are statistically insignificant (values within blue dashed lines). Such diagnostic tests were performed for all 66 of the available samples.

**Figure 7:**
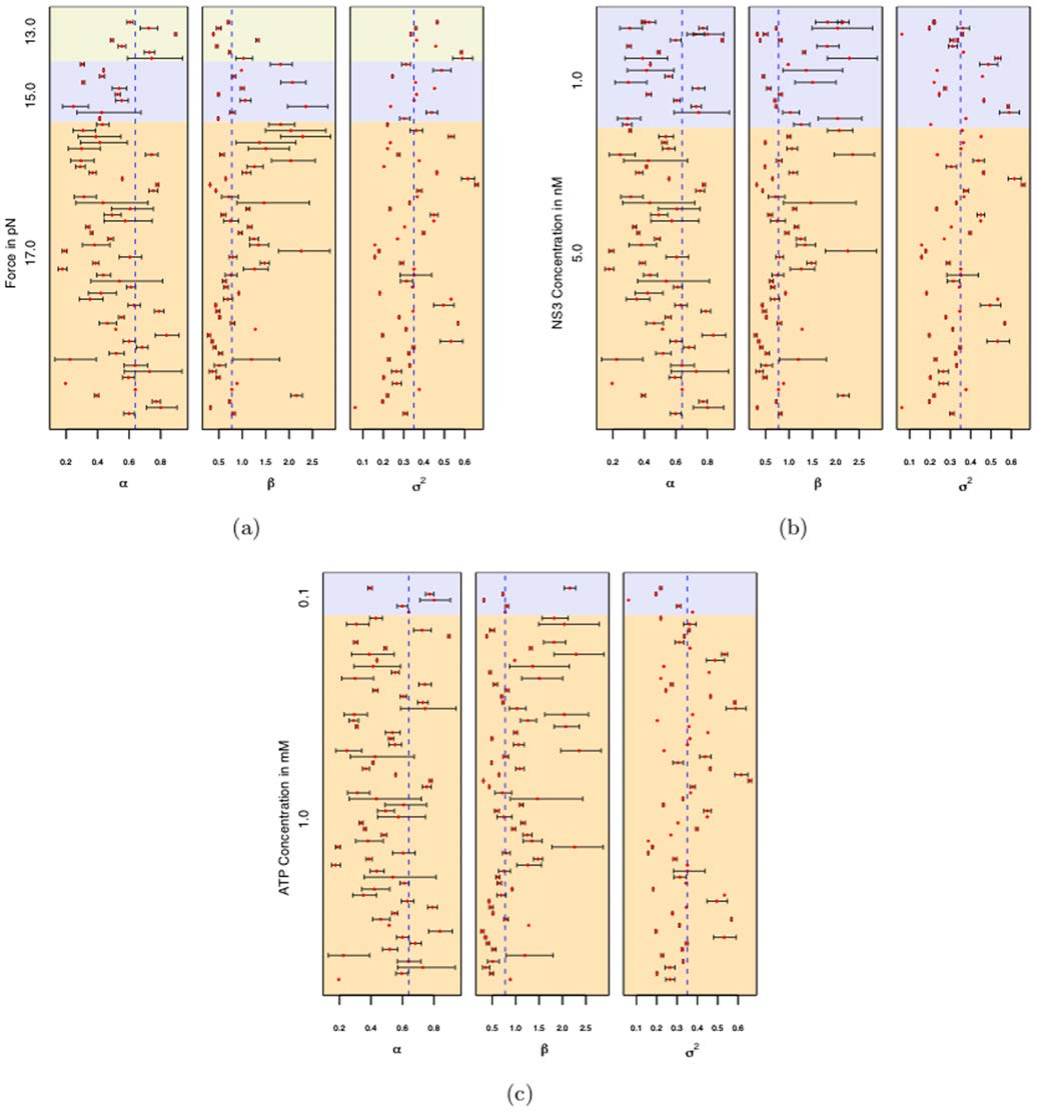
Estimates of parameters. *α*, *β* and *σ^2^* and their associated 95% confidence intervals. Also shown are the estimates for the various values of Force, ATP and NS3 concentrations. The dashed line indicates the weighted mean of the parameters.

Figure 7 shows the estimates of the parameters 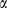, 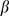 and 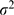 and their associated 95% confidence intervals. The plot also shows the estimates corresponding to the different values of the Force, ATP and NS3 concentrations. The parameter estimates seem to be independent of the force, ATP and NS3 concentrations. We computed a weighted mean of the parameters using as weights the inverse of the variances.(Fairly standard statistical procedure to use inverse of the variance as the weights. The idea is that the quantities with higher variance i.e. those that are less precise contribute less to the mean.) This is indicated by the vertical dashed line. The weighted estimates of 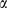, 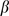 and 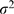 are 0.64, 0.77 and 0.35 respectively. Quantile -Quantile plots for the jump intervals like those in figure 5 were plotted with these new parameter values and they seem to satisfy the assumptions as before.

The gamma distribution with 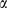 equal to one is a homogenous exponential distribution with rate 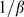. A renewal process with inter-jump interval following an exponential distribution is a poisson process. It is seen from figure 7 that in none of the 66 samples does the 95% confidence interval for 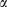 include the value 1. This suggests that it was inefficient to impose the assumption that the inter-jump intervals followed a poisson process as in [1].

### Pauses and Subpauses

Dumont et.al. [1] define pauses as periods of constant extension between steps. They also identify each step being composed of a number of substeps (this will be discussed below). The period of constant extension between these substeps is defined as a subpause. The estimation of the step locations using equation (3) and algorithms in [6] gives an estimate of the duration of the periods of constant extension. This leads to two questions:

1. Can the periods of constant extension be classified into pauses and subpauses? Are the two classes statistically distinguishable?
2. Are the pauses and subpauses independent of the applied force, NS3 concentration and ATP concentration?

### Pause-Subpause Classification

**Figure 8:**
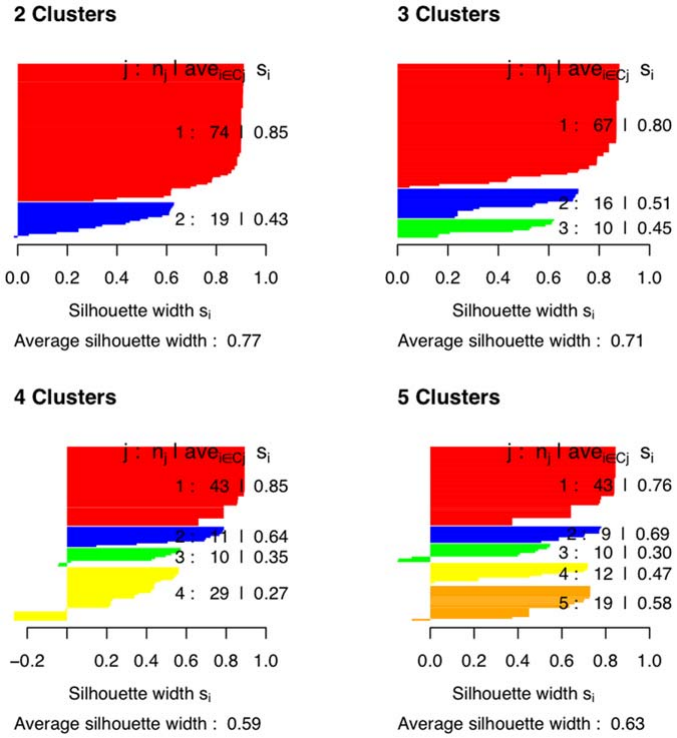
PAM Silhouette plot. Shows classification of periods of constant extension obtained for traces with 1 mM NS3, 1 nM ATP and a force of 13 pN.

Here we employ the classification algorithm Partitioning Around Meloid (PAM) [15] to see if the periods of constant extension can be classified into pauses and subpauses, i.e to see if a cluster of size two is the ideal classification. The output from PAM produces what is known as the silhouette width ranging from 0 to 1 for a given cluster size. The greater the value of the silhouette width, the better the classification. A silhouette width ranging between 0.7 and 1 indicates a very strong structure in the classification and between 0.5 and 0.7 indicates a reasonable structure in the classification. We first run PAM individually on the durations across different experiments. Cluster sizes of 2, 3, 4 and 5 were employed. The silhouette plot from one such analysis is presented in figure 8.

It can be seen from figure 8 that the average silhouette width is highest for the cluster of size 2 with an average of 0.77. This cluster shows that the subpause group (red) has a well pronounced structure while the pause group (blue) has a weaker structure. This could be due to the small number of pauses compared to subpauses. As the number of clusters is increased, the strength of some of the individual groups seems to increase, but the average silhouette width decreases. Thus a cluster of size two seems to do the best classification. This procedure was repeated for durations from other sets of experiments. Each of them consistently chose a cluster of size two with an average silhouette width ranging between 0.74 and 0.77. Thus a cluster of size two seems to be the ideal classification and we will refer to them as pauses and subpauses.

To avoid misclassification by sheer chance of the observed data we incorporate the PAM classification procedure into a leave-one-out algorithm. We leave one trial out and run PAM on the durations from other trials. Using the results from PAM, the durations from the left out trial are classified as a pause or a subpause. This procedure is repeated for every trial and a given duration is given the majority classification. This reduces the probability of misclassifying a given duration as pause or subpause. It also gives a measure of the sensitivity of a given duration to misclassification, i.e. in what proportion of the leave one out trials was a given duration classified differently from its final classification. A proportion of zero would suggest that a given duration is clearly a pause or a subpause. Higher proportions might indicate borderline values that could have been classified as a pause or a subpause depending on the draw of the data.

**Figure 9:**
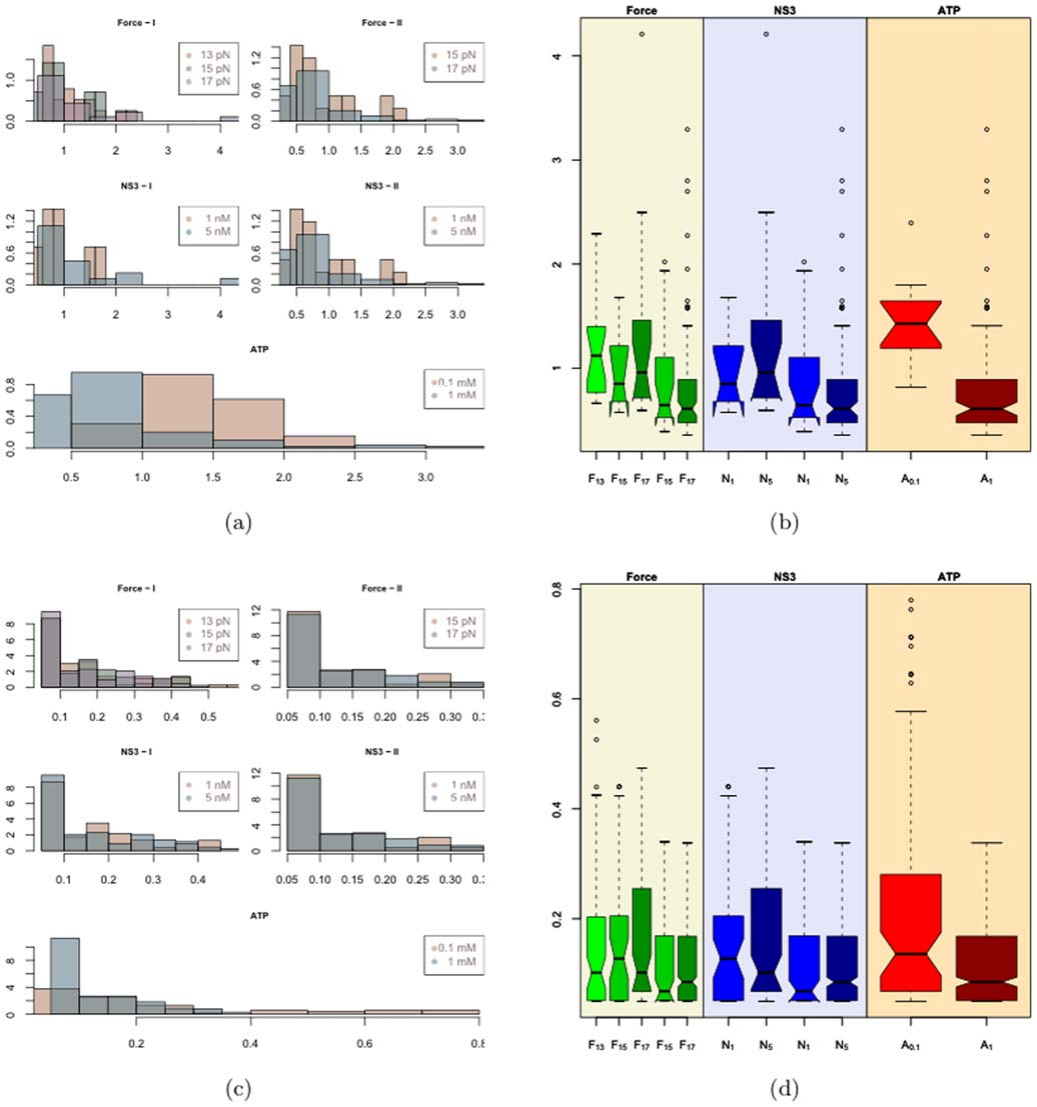
Histogram and Boxplots for Pauses and Subpauses.

**Figure 10:**
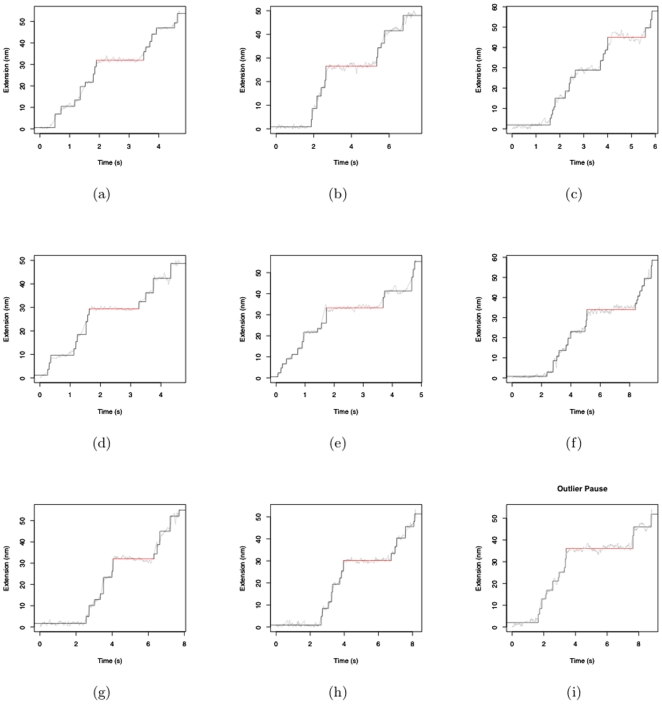
Outlier Pause. The gray line indicates the raw data. The black line indicates the fitted value. The red line indicates the outlier pause seen in figure 9D.

**Figure 11:**
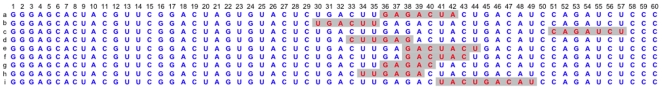
Outlier Pause. The RNA sequence corresponding to the outlier pauses. The gray box shows the area of interest i.e. the region at which pause durations were observed.

Figure 9 shows the empirical distribution of the pauses (a,b) and the subpauses (c,d) respectively. The histograms suggest the distribution is right skewed. The notches in the boxplots in figures 9 (b) and 9(d) seem to overlap suggesting that their median values might be similar at the 5% significance level except in the case of the ATP comparison boxplots. The difference is more pronounced in the case of pauses then the subpauses. The outliers in the subpauses are the borderline values suggested by PAM. It was observed from the leave one out PAM classifications that these subpauses were classified as pauses in about a third of the trials. The outliers in the pauses in figure 9(b) are shown in figure 10 as the horizontal red lines. The black lines show the fitted values superimposed on the raw data represented by the gray lines. Diagnostic checks like the one in figure 5 reveal no abnormalities. The consistency of their pattern of occurrence suggests that it might not be appropriate to dismiss them as outliers. They seem to occur consistently either between 2 and 6 seconds or between 4 and 8 seconds. Pauses are the time duration that it takes to break the hydrogen bonds before proceeding with the unwinding process. Thus in the time it takes to break a bond the apparatus records constant extension. A relevant question that merits further investigation with regard to these outlier pauses is, are there particular sequences of RNA that are more difficult to unwind than others? To address this question, the numbers of base pairs of RNA unwound just before the pause and just after the pause were calculated. This segment is shown in figure 11 by the sequence in the gray shaded area. It is seen that except for outlier C in figure 10 the others are overlapping areas in the same region of the sequence. These outliers also account for those that were observed in the QQ-plots in figure 5. Such dependence of the pauses and the jumps on the base sequence have been reported in [7], [8] with respect to DNA unzipping.

Hypothesis tests to test the effect of force, NS3 and ATP concentration on pause subpause durations suggests that the pause and subpause durations might be independent of the force applied and the NS3 concentration. It also showed that the difference is statistically significant in the case of ATP concentrations suggesting that the pause-subpause durations might be dependent on ATP concentration. This can explain the outliers in the QQ-plot in figure 5 where lower concentration of ATP had more outliers. Dumont et.al. [1] explain this on the basis of the fact that ATP is needed to provide the energy required to break the hydrogen bonds. Thus decrease in the concentration of ATP appears to increase the waiting time for the next molecule of ATP to aid the unwinding process.

As the pauses and subpauses seem to be independent of the force applied and the NS3 concentration, one can pool the data and get ATP specific estimates of the pause and subpause durations. The estimates are shown in Table 1. From table 1 it seems that the pause subpause durations decrease with increase in ATP concentration.

**Table 1:** Pause - Subpause estimate across ATP concentration.

ATP (mM)	Pause			Subpause		
	Mean	SE	95% CI	Mean	SE	95% CI
0.1	1.46	0.11	(1.24, 1.67)	0.26	0.02	(0.18, 0.28)
1	0.9	0.04	(0.81, 0.99)	0.12	0.003	(0.11, 0.13)

### Steps

In the previous section, the periods of constant extension were classified as pauses and subpauses using the partitioning around medoids (PAM) algorithm. Dumont et.al. [1] define steps as the extension of RNA between two pauses. While this could be a single step, in most cases it appears to be composed of a series of substeps. The periods of constant extension between these substeps are the subpauses. Having identified the pauses, we can now infer the steps and the number of substeps they are composed of.

### Number of substeps per step

Here we look at the distribution of the number of substeps that compose a step i.e. the number of substeps between two pauses and the effect of force applied, NS3 and ATP concentration on it.

Hypothesis tests to study the effect of force, NS3 and ATP concentrations suggests that the number of substeps per step is independent of the force applied, NS3 and ATP concentrations. As the number of substeps per step seems independent of the various factors, one can get a better estimate by pooling the data from the various experiments. The estimate from the pooled analysis is shown in table 2. Thus one can infer that the number of substeps per step might be independent of the force applied, NS3 and ATP concentrations and one can expect on average 4 substeps per step. Dumont et.al. [1] report between 2 and 5 substeps per step and hypothesize on 3 substeps per step based on a poisson analysis.

**Table 2:** Estimate: Number of substeps per step.

Number of Substeps per Step		
Mean	SE	95% CI
4.1	0.2	(3.6, 4.5)

### Step Size

We will now analyze the distribution of the step size expressed in base pairs. The extension between two pauses expressed in nanometers can be converted at the given force to base pairs using the worm-like chain model (WLC) [16] given by
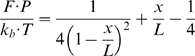
(5)where

1. 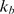 is the Boltzmann constant 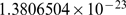 Joules/Kelvin.
2. 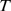 is the temperature in Kelvin.
3. 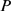 is the persistence length of the polymer. The persistence length is a property quantifying the stiffness of a long polymer. Heuristically, for polymers shorter than the persistence length, the molecules behave like a rod and for polymers longer than the persistence length the properties can be described only statistically.
4. 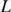 is the Contour length of the polymer. The contour length of a polymer is its length at the maximum physically possible extension.

Hypothesis tests to study the effect of force, NS3 and ATP concentration on step size suggests that the mean step size might be independent of the force applied, NS3 and ATP concentrations. Thus one can pool the values to get a better estimate of the step size. The estimate from the pooled analysis is given in table 3.

**Table 3:** Estimate: Step size.

Step Size (bp)		
Mean	SE	95% CI
16.2	0.7	(14.7, 17.6)

Dumont. et.al. [1] report a mean step size of 11 base pairs with standard error of 3 base pairs which puts our point estimate within their 95% confidence intervals. Earlier studies [5] report the mean size as 18 base pairs with a standard error of 2 base pairs. Our estimate seems to fall right in between with respect to the mean, but has a much smaller variance. An estimate of the median value and its 95% confidence interval given in table 4 seems to be more in accordance with the results in [1].

**Table 4:** Estimate: Step size.

Step Size (bp)		
Median	SE	95% CI
11.37	0.34	(10.69, 12.05)

### Substeps

In this section we look at the distribution of the substep sizes. Steps that occur between two pauses are referred to as substeps. Knowing the location of the pauses we can extract the substeps. If there is only a single step between two pauses we will not include it in computing the substep statistics. It will be considered a single step.

Hypothesis tests to study the effect of force, NS3 and ATP concentration on substep size seems to suggest that the substep size is independent of force, NS3 and ATP concentration. Pooling the data, an estimate of the substep size is given in table 5.

**Table 5:** Estimate: Substep size.

Substep Size (bp)		
Mean	SE	95% CI
3.7	0.05	(3.6, 3.8)

### Stepping Velocity

The stepping velocity is the velocity of the NS3 molecule as it unwinds a set of base pairs constituting a step. Dumont et.al. [1] define the stepping velocity as the slope of the unwinding trace between two pauses. In this work the estimation of velocity 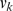 of the 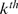 step is directly incorporated into equation (3). There is a difference in the definition of velocity in this work and that of [1] as a direct consequence of the method used to model the unwinding process. Dumont et.al. first detect steps and then use a smaller running window within the step to detect substeps. Using their terminology, every step detected using equation (3) is a substep and we define a step as comprising of those substeps occurring between pauses. If there exists only one substep between two pauses that will be referred to as a step. Thus it is the substeps that occur naturally and a step is just a definition i.e. a collection of substeps between two pauses. Our method estimates the stepping velocity of each of these substeps. Though Dumont et.al. [1] report as stepping velocity the slope of the unwinding trace between two pauses, a relevant question to pose is whether such a velocity exists, as there does not exist a natural step movement between pauses except when there is just one substep between pauses. Thus the results from [1] may not be comparable to one in this work due to the way in which stepping velocity is defined. We propose that the stepping velocity be defined as the slope between two subpauses or between a pause and a subpause.

**Figure 12:**
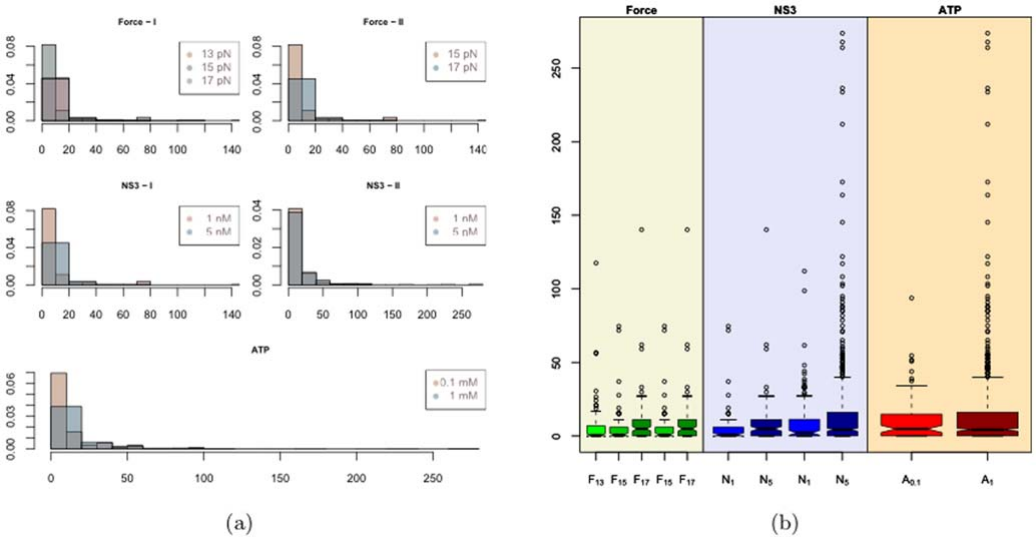
Histogram and Boxplots of estimated Stepping velocities.

We now analyze the distribution of the stepping velocity and the effects of the force applied, NS3 and ATP concentrations. Figure 12 shows the histograms and the notched boxplots of the estimated values of the stepping velocity. The histogram shows the distribution to be right skewed in all the experiments. The outliers in the boxplot reveal the extent to which the distribution is right skewed. In fact 10% of the observation in each category is an outlier. The maximum velocity that the measuring device could follow was 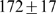 nm/s, the upper limit of which translates to about 230, 222 and 216 bp/s at 13, 15 and 17 pN force respectively. All but five of the estimated velocities were within this limit with the maximum being 273 bp/s.

**Figure 13:**
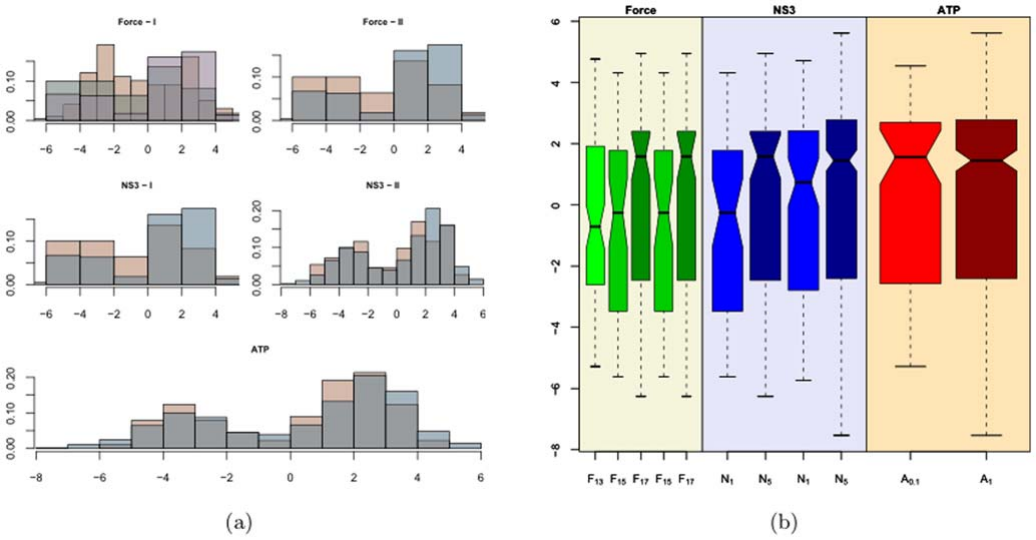
Histograms of Log transformed Stepping velocities.

**Figure 14:**
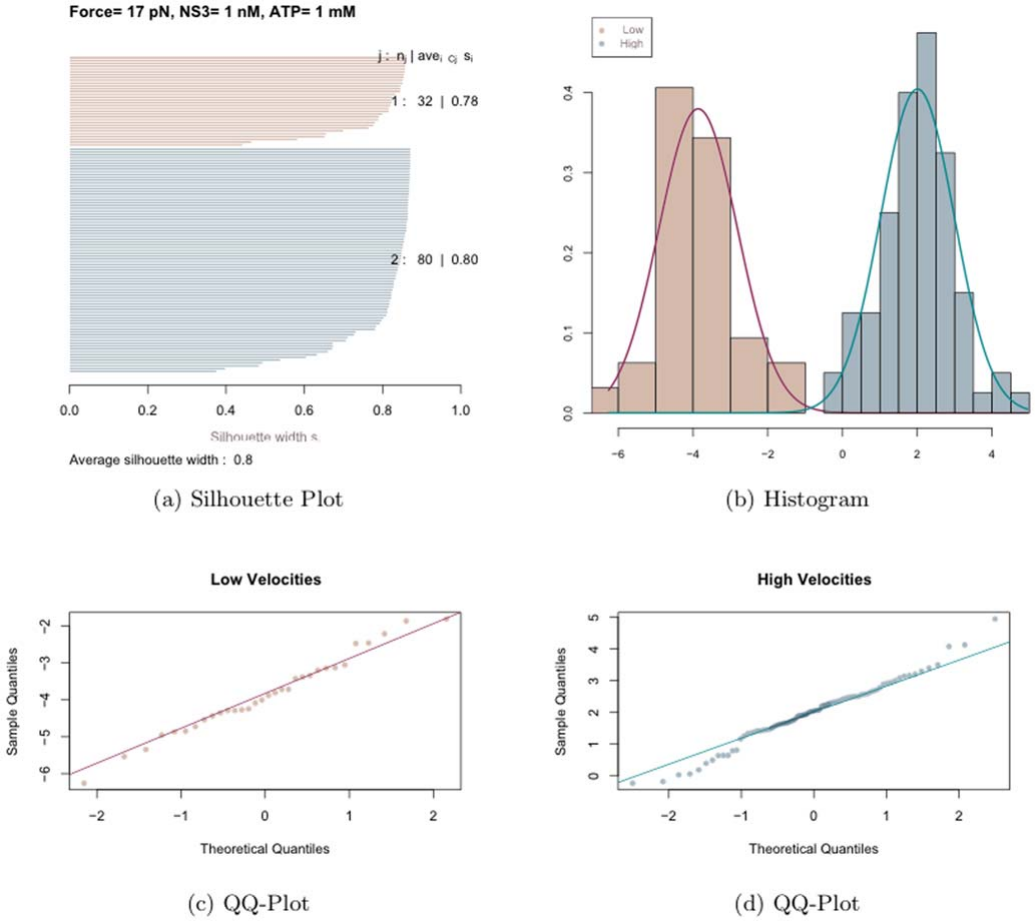
Classification of Log transformed velocities. (a) shows the Silhouette plot from the classification, (b) shows the histograms of the two velocities with the normal density overlaid, (c) and (d) show the QQ-plots of the velocities against the theoretical quantiles of the Normal distribution.

Figure 13 shows the histograms and the boxplots of the log transformed stepping velocities. One can see from the boxplots that the variance in the velocity has been stabilized and there are no longer any outliers. Though it may not be evident from the boxplots, the histograms (especially the plots on the right and bottom) suggest a bimodal distribution for the log transformed velocities. This leads to the question: do there exist two kinds of stepping velocities like the two kinds of pauses seen before. To see if the velocities can be classified into two distinct groups we run the clustering algorithm partitioning around medoids (PAM) on the velocities from each of the experiments. The algorithm was run for cluster sizes 2, 3, 4 and 5. A cluster of size two was consistently chosen across all the experiments with average silhouette widths ranging between 0.7 and 0.8 indicating a strong structure for the two groups. The leave one out algorithm incorporating PAM was run to classify the velocities into two groups. We refer to them as the low and high velocity groups. Figure 14 shows the results from the classification of the log transformed velocities into two groups from one set of experiments. Figure 14 (a) shows the silhouette plot indicating the cluster structure obtained from PAM. The silhouette widths of 0.78 and 0.8 suggest the existence of two clusters with very strong structures. Figure 14 (b) is the plot of the histogram of the two groups with the normal density curve overlaid. It shows the existence of two distinct distributions. Figures 14 (c) and (d) are the quantile-quantile plots of the sample quantiles of the log transformed velocities against the theoretical quantiles of the normal distribution. It can be seen that both the low and high velocities can be well approximated by a normal distribution. Such inferences were drawn from all the experiments. Thus one might infer that the stepping velocities can be classified into low and high and that each of them follow a log-normal distribution. This explains the extreme right skew seen in figure 12.

Hypothesis tests to study the effect of force, NS3 and ATP concentration on velocity seems to suggest that the velocities both low and high are independent of force, NS3 and ATP concentration. Thus one can pool the values to obtain a better estimate. The estimate from the pooled computation is shown in table 6 (log transformed velocities) and table 7 (velocities in the original scale).

**Table 6:** Estimate: Log-Velocity. Mean and the 95% CI for the log-transformed values of velocity.

Low Velocity log (bp/s)		
Mean	SE	95% CI
−3.2	0.07	(−3.3, −3.0)
High Velocity log(bp/s)		
Mean	SE	95% CI
2.3	0.05	(2.2, 2.4)

**Table 7:** Estimate: Velocity. Mean and the 95% CI for the velocity in the original scale obtained by exponentiating values in table 6.

Low Velocity bp/s	
Mean	95% CI
0.04	(0.03, 0.05)
High Velocity bp/s	
Mean	95% CI
10	(9, 11)

It was observed that the high velocity is the dominant one occurring in 60% of the cases. This was significant as the hypothesis of equal proportions of low and high velocities were rejected using a binomial model. It is now of biological interest to understand what causes this difference in the velocities one of which is nearly 250 times the other.

## Discussion

The results presented in the above analysis can be summarized as follows:

1. The intervals between jumps is described well by a gamma distribution, the parameters of which seem to be independent of the force, NS3 and ATP concentrations.
2. The periods of constant extension can be classified into pauses and subpauses. They seem to be ATP dependent with the durations increasing with decreasing ATP concentration.
3. The number of substeps per step, the substep and the step size, all seem to be independent of force, NS3 and ATP concentrations.
4. The stepping velocity can be classified into low and high velocities. Each following a log-normal distribution.

There is an overall agreement between the inferences drawn in [1] and this work regarding the RNA unwinding characteristics. It is worth noting that this work makes only two assumptions-the intervals between the jumps being gamma distributed and the noise being normally distributed. These are assumptions that one can validate after the model is fit. This work avoids assumptions or conditions that either have no basis nor can be verified after model fit. Two characteristics that are worth exploring are

1. Step size: Both [1] and this work conclude that the step size is independent of the force, NS3 and ATP concentrations but differ in the average step size reported i.e. 11 bp in [1] and 16 bp in our work. The biologists hypothesize that a 11 bp step size might make more biological sense from the viewpoint of the chemical structure of both RNA and NS3. Due to the right skewed distribution of the step size the median might be a more appropriate statistic to consider. In this work the median step size does appear to be in agreement with the results in [1]. To address this disparity, a future work would be to explore the outlier cases described in this work. What leads to the higher number of substeps between two pauses? Are such behaviors just anomalies? If so, does their exclusion lead to an agreement between step size between the two studies with respect to the mean step size?
2. Stepping Velocity: There are some basic differences in which the velocities are computed in the two studies and as such are incomparable. Unlike [1] our work suggests that the velocity might be independent of ATP concentration, but one should note that our work classified the velocities into low and high. Further experimental studies might be required to understand the phenomenon. Another area of interest is to study the relation between pauses and their occurrence in relation to the RNA sequence as discussed in this work.
